# Selective Detection of Cu^+^ Ions in Live Cells via Fluorescence Lifetime Imaging Microscopy

**DOI:** 10.1002/anie.202109349

**Published:** 2021-09-17

**Authors:** Martin Priessner, Peter A. Summers, Benjamin W. Lewis, Magdalena Sastre, Liming Ying, Marina K. Kuimova, Ramon Vilar

**Affiliations:** ^1^ Department of Chemistry Imperial College London White City Campus London W12 0BZ UK; ^2^ Department of Brain Sciences Imperial College London Hammersmith Campus London W12 0NN UK; ^3^ National Heart and Lung Institute Molecular Sciences Research Hub White City Campus Imperial College London London W12 0BZ UK

**Keywords:** copper(I), FLIM ^.^, fluorescence, lifetime, TCSPC

## Abstract

Copper is an essential trace element in living organisms with its levels and localisation being carefully managed by the cellular machinery. However, if misregulated, deficiency or excess of copper ions can lead to several diseases. Therefore, it is important to have reliable methods to detect, monitor and visualise this metal in cells. Herein we report a new optical probe based on BODIPY, which shows a switch‐on in its fluorescence intensity upon binding to copper(I), but not in the presence of high concentration of other physiologically relevant metal ions. More interestingly, binding to copper(I) leads to significant changes in the fluorescence lifetime of the new probe, which can be used to visualize copper(I) pools in lysosomes of live cells via fluorescence lifetime imaging microscopy (FLIM).

## Graphical abstract

Herein we report a new fluorescent probe that upon binding to copper(I) (but not any other physiologically relevant metal ions), displays an intensity switch‐on as well as a significant change in its fluorescence lifetime. The latter has allowed us to visualize copper(I) in live cells via fluorescence lifetime imaging microscopy.

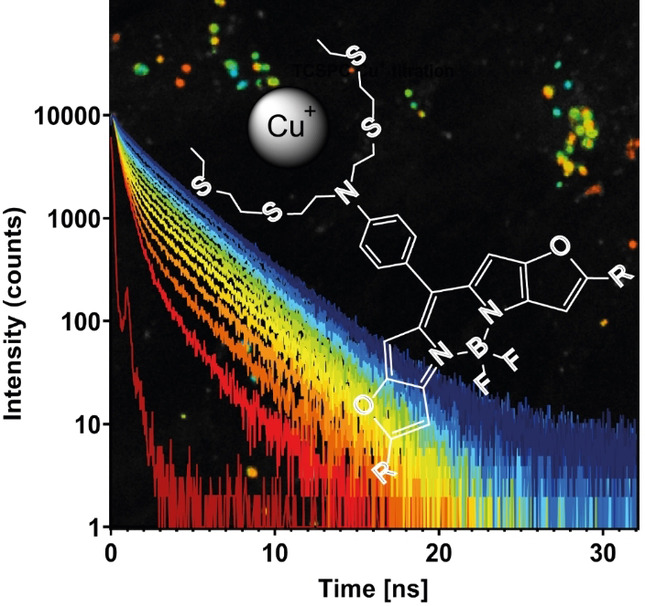

Copper is an essential trace element for living organisms. It can cycle between copper(II) and copper(I), with the latter being the predominant oxidation state present under physiological conditions. In a cellular environment copper ions can coordinate to a range of nitrogen‐ and sulphur‐based ligands.^[1]^ It participates in single‐electron transfer reactions and, therefore, is involved in a wide range of biological processes including respiration, free radical scavenging, iron metabolism, connective tissue biogenesis and synthesis of neuropeptides.[^2^, ^3^, ^4^] It is tightly regulated by a system of intracellular chaperones and transporters (e.g. CTR‐1, ATOX‐1, GSH)^[5]^ that prevent the accumulation of free copper ions. Cellular copper ions are generally found in two forms: either in a static pool, where the metal is tightly bound to proteins and other macromolecules, or in a labile pool, where it is weakly bound to cellular ligands and, hence, mobile.^[6]^ Mismanagement of cellular copper pools can result in oxidative stress with the formation of reactive oxygen species (ROS) through Fenton‐type reactions.^[7]^

Due to the important biological roles that copper plays, its misregulation can have significant implications for health and disease. Excess copper causes toxicity and compromises the redox homeostatic state of cells. The toxicity of a copper overload becomes particularly clear in Wilson disease, a pathological condition caused by a mutation in the *ATP7B* gene (which encodes a copper‐transporting ATPase), causing a build‐up of copper in the liver and several other tissues.^[8]^ On the other hand, Menkes disease is a genetic disorder associated with impaired copper efflux from enterocytes into the blood and inadequate transport of copper to the brain.^[9]^ Furthermore, copper is believed to play a crucial role in the progression of several neurogenerative diseases such as Parkinson's,^[10]^ Alzheimer's^[11]^ and Huntington's^[12]^ diseases.

Copper's biological relevance has motivated the development of techniques to measure its cellular levels. These include inductively coupled plasma mass spectrometry (ICP‐MS),^[13]^ X‐ray fluorescence microscopy (XFM)^[14]^ and nano‐secondary ion mass spectrometry (Nano‐SIMS),^[15]^ amongst others. While these techniques have provided important insights into the biology of copper, they are not capable of measuring and tracking it in living cells. Therefore, there is significant interest in the development of fluorescent probes that can selectively respond to copper ions, enabling its direct visualisation in live cells. Over the last two decades several such fluorescent probes for copper(I) have been reported in the literature.[^16^, ^17^, ^18^, ^19^, ^20^, ^21^] Most of these probes turn‐on their fluorescence intensity upon binding to copper(I) via a photoinduced electron transfer (PET) process or a related charge transfer (CT) pathway.^[22]^ However, intensity‐based probes have the disadvantage that they are concentration dependent, that is, their signal is strongly dependent on probe uptake to a particular intracellular location.

An attractive alternative to intensity‐based probes are fluorophores that change their fluorescence lifetime upon binding to a target analyte. Since this parameter is concentration independent, it can provide a more realistic map of the cellular distribution of a target of interest. Thus, by using fluorescence lifetime imaging microscopy (FLIM), in conjunction with a fluorophore with lifetime‐sensitivity to copper(I), it should be possible to detect and image true levels of copper(I), even in the presence of competing species.

FLIM has been previously used to investigate the behaviour of intracellular copper(II).[^23^, ^24^, ^25^, ^26^, ^27^] In contrast, to the best of our knowledge there are no previous studies where this approach has been employed to visualize copper(I) in live cells. Herein we report a new fluorescent probe (**FLCS1**, Scheme 1), which switches‐on its intensity in the presence of copper(I). Additionally, it displays a distinct fluorescence lifetime change upon copper(I) binding, which can be used to visualize this metal ion in live cells via FLIM.

**Scheme 1:**
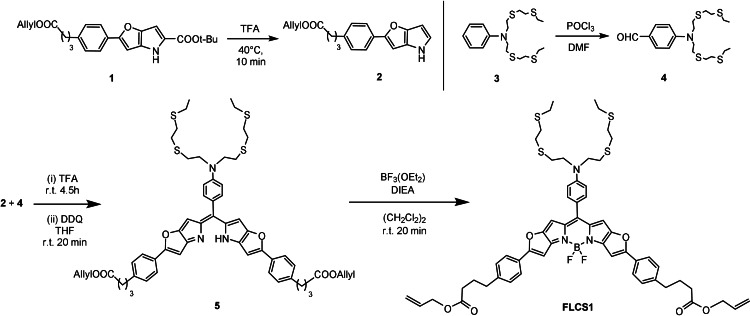
Synthetic scheme for the preparation of the new BODIPY‐based optical probe **FLCS1** (for full reaction Scheme, see Supporting Information).

The design of our new probe (**FLCS1**, see Scheme 1) is based on linking the known NS_4_ copper(I) receptor pioneered by Chang et al.^[28]^ with a highly conjugated BODIPY‐based fluorophore, which displays good photostability, high quantum yield and a red shifted emission, in the spectral range suitable for cell and tissue imaging.^[29]^ We expected PET from the electron‐rich NS_4_ chelating fragment to the BODIPY core to quench its emission in the absence of copper(I).^[30]^ Fluorescence should switch‐on upon copper(I) binding, due to a reduction of the electron‐donating ability of the NS_4_ receptor towards the BODIPY fluorophore.

**FLCS1** was obtained via the synthetic pathway shown in Scheme 1. The tetrathia‐aldehyde building block **4** and the BODIPY precursor **2** were synthesised following previously reported procedures (see Supporting Information for full reaction Scheme and experimental details).^[29]^ To synthesise the new compound **5**, it was first necessary to deprotect **1** with trifluoroacetic acid (TFA) to yield **2**, which was immediately reacted with compound **4**. This was followed by solvent exchange (to THF) and addition of DDQ yielding **5**. In the final step, a crude sample of **5** was reacted with DIEA and BF_3_⋅Et_2_O to yield **FLCS1** which was purified by column chromatography and preparative TLC. The new probe was characterised by ^1^H and ^13^C NMR spectroscopy and MALDI‐TOF (see Supporting Information for spectroscopic data).

With the new BODIPY‐based probe in hand, a series of studies were performed to establish its response to copper(I). **FLCS1** displayed characteristic optical features of a BODIPY chromophore with extended conjugation, showing the main absorption band at 646 nm (*ϵ*=2.48×10^5^ M^−1^ cm^−1^) and a broad shoulder at 594 nm (*ϵ*=8.07×10^4^ M^−1^ cm^−1^), Figure 1 a. Addition of copper(I) to a methanolic solution of **FLCS1** leads to a slight bathochromic shift (4 nm) of the *λ*_max_ in the UV–Vis spectrum. Importantly, addition of copper(I) to **FLCS1** switches on its fluorescence (Figure 1 b). While a methanolic solution of the probe in the absence of copper(I) is practically non‐fluorescent (*Φ*=0.032), increasing amounts of copper(I) lead to a clear enhancement of the emission band centred at 660 nm.

**Figure 1:**
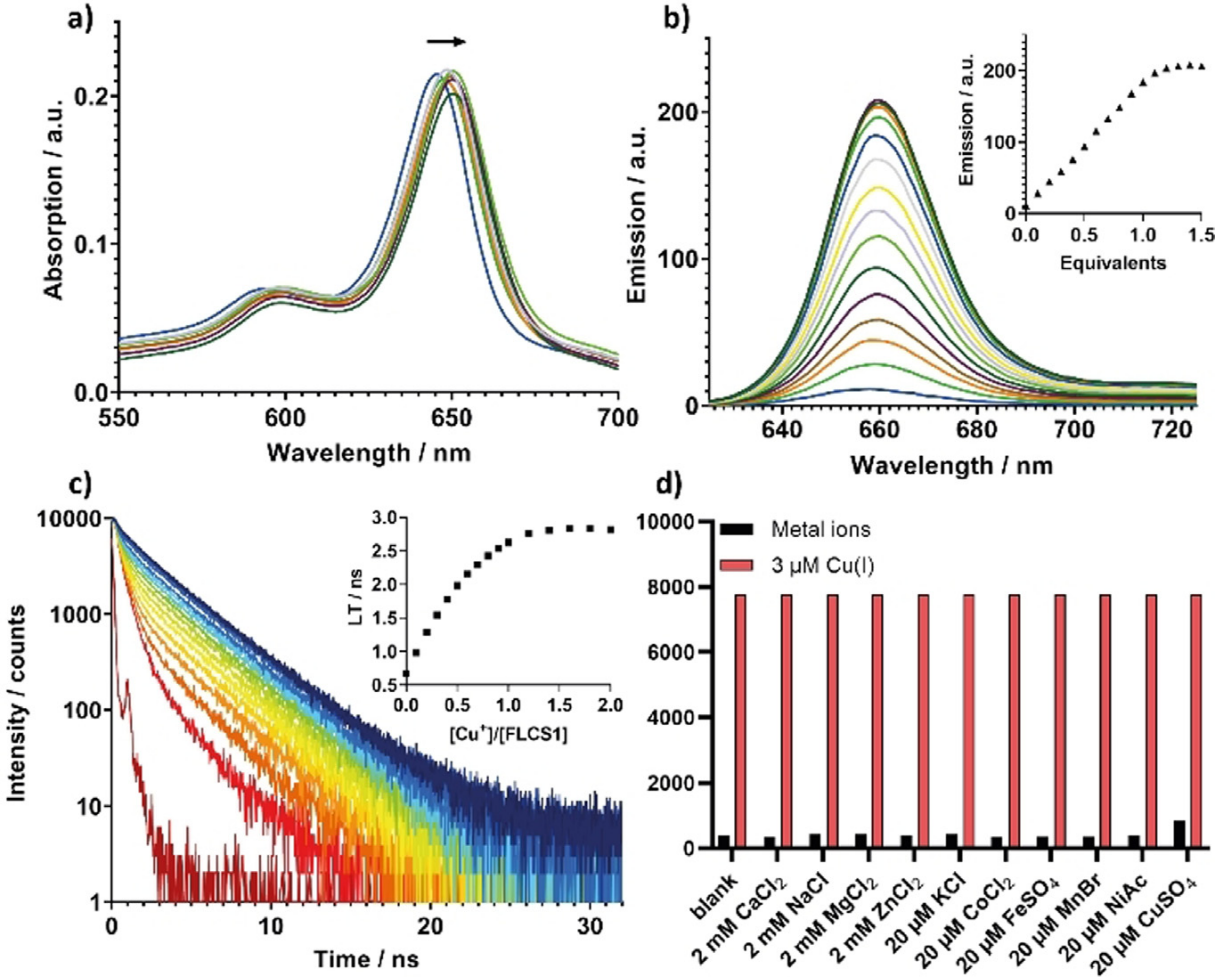
Spectroscopic studies of **FLCS1** (1 μM) in methanol upon addition of increasing amounts of copper(I), up to 1.6 equivalents. a) UV–Vis spectra; b) Fluorescence spectra (λ_ex_=610 nm); inset shows the binding plot recorded at the fluorescence peak maximum, λ_emis_=660 nm. c) Time‐resolved fluorescence decays of **FLCS1** (λ_ex_=630 nm, λ_emis_=660±10 nm); instrument response function (IRF) is shown in dark red; inset shows calculated intensity‐weighted average lifetime vs. Cu^+^/**FLCS1** ratio. d) Response of **FLCS1** (1 μM in methanol) to the addition of different metal ions with bars representing the integrated fluorescence response of the probe. Black bars represent the response to the addition of excess metal ions (2 mM Ca^2+^, Mg^2+^, Zn^2+^ and Na^+^; 20 μM Co^2+^, Fe^2+^, Mn^2+^, Ni^2+^, Zn^2+^, K^+^ and Cu^2+^); red bars represent the response to the subsequent addition of Cu^+^ (3 μM) to the corresponding metal ion solutions.

Saturation was reached after the addition of approximately one equivalent of copper(I), at which point the fluorescence intensity increased by ca. 20‐fold (*Φ*=0.66). The one‐to‐one binding stoichiometry between **FLCS1** and copper(I) was confirmed by the method of continuous variation (i.e. a Job plot), which showed a clear maximum at 0.5 mol fraction (see Figure S4a). The K_d_ between **FLCS1** and copper(I) was determined in a buffered thiourea solution (in methanol) following a previously reported procedure (see Figure S4b,c),[^31^, ^32^] giving a value of 2.6×10^−9^ M (this value cannot be directly compared to the K_d_ values previously reported for similar copper(I) probes since different solvents were used). To assess the reversibility of the process, the **FLCS1**‐Cu^+^ complex was treated with 2 equivalents of PSP‐2, which is known to have a very high affinity to copper(I).^[33]^ This led to a decrease of the fluorescence intensity down to the basal level, observed for the free probe. Addition of excess copper(I) to this solution led to a complete restoration of the fluorescence intensity, initially observed for the **FLCS1**‐Cu^+^ complex, confirming that the binding event is reversible (see Figure S5).

Interestingly, the fluorescence lifetime of **FLCS1** increased significantly upon binding to copper(I) (see Figure 1 c). The time‐resolved fluorescence traces were best fitted using a biexponential decay function, with two lifetime components (τ_1_=0.39 ns and τ_2_=3.08 ns) extracted from a global fit of all decays recorded upon the addition of copper(I). The full set of fitted data is presented in the Supporting Information (Figure S6). We assign the short lifetime component (τ_1_=0.39 ns) to free **FLCS1** and the long lifetime component (τ_1_=3.08 ns) to copper‐bound **FLCS1**, which is supported by the change in fitted amplitudes of the two components upon copper(I) titration (Supporting Information, Table S1 and Figure S7).

To determine the selectivity of **FLCS1** for copper(I), we studied the fluorescence response of the probe upon addition of physiologically relevant concentrations of various metal ions usually present in the cell. Pleasingly, no switch‐on effect of the fluorescence of **FLCS1** was observed upon addition of 2 mM CaCl_2_, NaCl, MgCl_2_ and ZnCl_2_, nor upon the addition of 20 μM concentrations of KCl, CoCl_2_, FeSO_4_, MnBr_2_, Ni(OAc)_2_, and CuSO_4_. A very slight increase was observed upon addition of 20 μM copper(II), which is consistent with previously published literature on probes with the same NS_4_ receptor (see Figure 1 d).[^32^, ^34^]

Having established the excellent in vitro copper(I)‐sensing capabilities of **FLCS1**, we investigated its cellular properties using neuroblastoma SH‐SY5Y cells. Cells were incubated with a 60 nM solution of **FLCS1** in the presence of 0.2 % lipofectamine in DMEM for 24 hours at 37 °C. Lipofectamine was needed for cellular uptake of **FLCS1** since this probe has poor water solubility and is not readily cell permeable. As can be seen in Figure 2, under these conditions the probe is taken up by cells and localises throughout the cytoplasm in specific cellular compartments with punctate distribution. After incubation with **FLCS1** cells showed to be fully viable, growing and replicating for over 48 hours (see Figure S8). Co‐staining experiments with Lysotracker Green (a well‐established dye for staining lysosomes) showed close colocalization of both probes (Figure 2). To confirm that the probe is not sensitive to acidic pH typically found in lysosomes, cells were treated with bafilomycin A1, which stops lysosomal acidification, thus increasing the pH. Pleasingly, confocal microscopy showed that this treatment did not alter the fluorescence intensity coming from **FLCS1** localised in lysosomes suggesting that these changes in pH do not affect the probe (see Figure S11).

**Figure 2:**
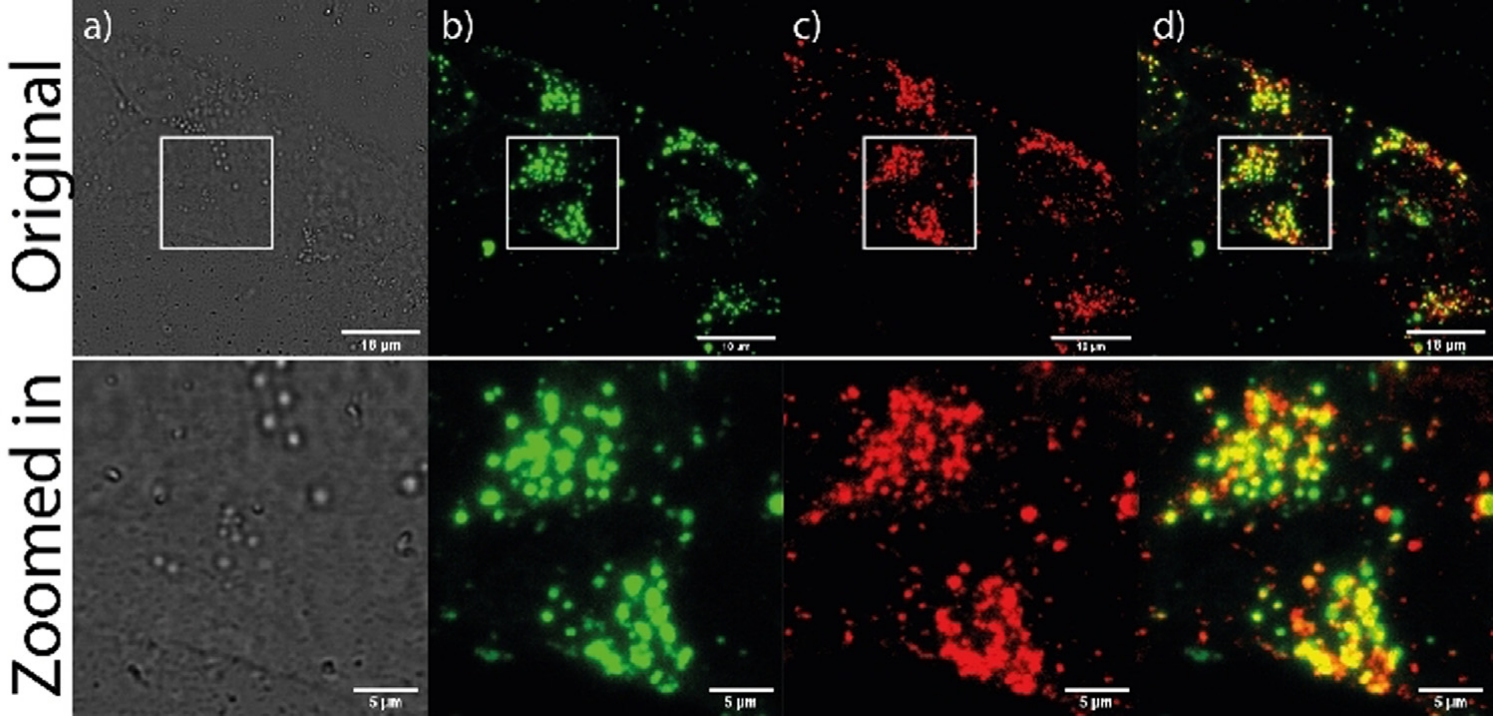
Colocalization of **FLCS1** with LysoTracker Green in SH‐SY5Y cells. Cells were incubated with 60 nM **FLCS1** with 0.2 % Lipofectamine for 24 h in DMEM and LysoTracker Green was used at 50 nM concentration in DMEM for 15 minutes before imaging. Images were recorded at 63x magnification with zoomed‐in sections highlighted by white boxes; the zoomed in sections are shown in the bottom row (a) brightfield image; b) imaging **FLCS1** (l_ex_=635 nm; l_emis_=645–770 nm); c) imaging LysoTracker Green (l_ex_=500 nm; l_emis_=510–530 nm); d) overlayed images b) and c), showing colocalization of LysoTracker Green with **FLCS1** (scale bare 5 μm).

In recent years, a growing body of evidence has indicated that lysosomes, which are involved in the degradation and recycling of cellular waste and energy metabolism,[^35^, ^36^] also play a vital role in the regulation of transition metal homeostasis including copper ions.[^37^, ^38^] Our microscopy studies are consistent with this theory and indicate the presence of copper(I) ions in lysosomes, since the fluorescence of **FLCS1** is clearly switched‐on in these organelles. However, as discussed above, intensity‐based probes are not concentration‐independent and, thus, it is not possible to assess based on the intensity alone, whether the high fluorescence observed is indeed due to the presence of large pools of copper(I) in lysosomes, or simply a consequence of high accumulation of the probe in this organelle. To address this, we recorded fluorescence lifetime imaging microscopy (FLIM) images of SH‐SY5Y live cells incubated with **FLCS1**. Since the cell‐free assays discussed above showed that **FLCS1** displays significantly longer fluorescence lifetimes when bound to copper(I) as compared to the metal‐free probe (see Figure 1 c), FLIM should provide an unambiguous confirmation of the presence of copper(I) in lysosomes.

The FLIM images (see Figure 3 a for a representative example) show biexponential time‐resolved decays (Figure 3 g) of **FLCS1** in cells, which is consistent with the presence of a mixture of bound and unbound probe, as in the cell‐free data (see Figure 1 c and Supporting Information, Figure S6). Fitting the images resulted in cellular distribution of τ_1_ centred around 1.1 ns and τ_2_ around 2.6 ns (see Figure S9g and i). These values deviate slightly from the observed in vitro values for **FLCS1** (copper‐free τ_1_=0.39 ns, copper‐bound τ_2_=3.08 ns). This difference could be due to the complex intracellular environment, as compared to the cell‐free assays (e.g. crowding, viscosity, polarity, refractive index). Indeed, we established that in vitro the probe is affected by various parameters; for example, adding increasing amounts of water to methanolic solutions of **FLCS1** decreases its fluorescence lifetime; increasing viscosity or decreasing the pH shifts the fluorescence lifetime of the probe to slightly lower values (see Figure S10). It is important to emphasise that none of these changes are large, but together with other factors such as the differences in the refractive index, they are sufficient to explain the small discrepancy in the lifetime values between the in vitro and in cellulo data.

**Figure 3:**
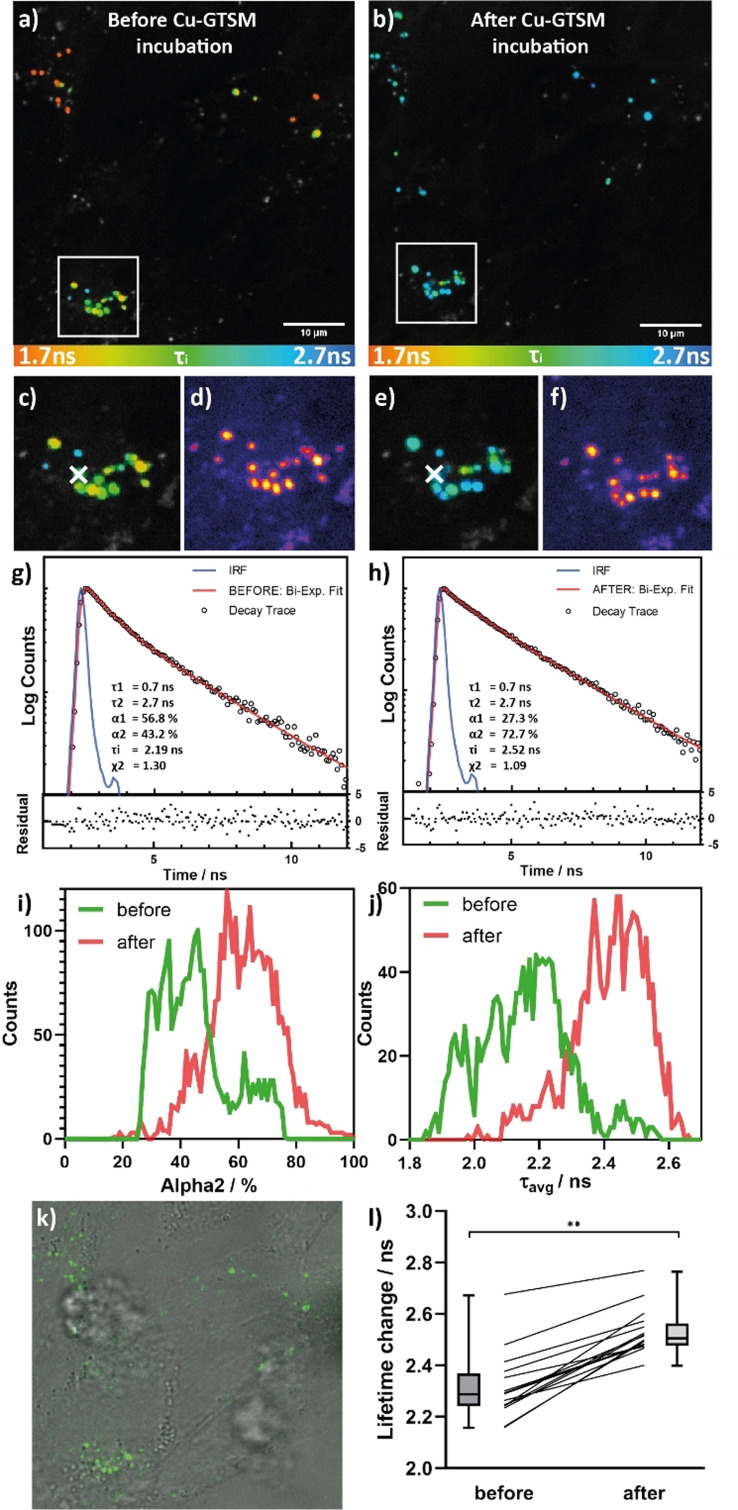
FLIM imaging of SH‐SY5Y cells treated with **FLCS1**. FLIM data recorded before (a, c, d) and after (b, e, f) incubation with 5 μM Cu‐GTSM for 20 min; a, b, c, e show the lifetime maps (τ_avg_); d) and f) show fluorescence intensity images; c, d, e, f show the zoomed in sections highlighted by white squares in (a) and (b); g) and h) show typical time‐resolved fluorescence decay curves from the regions indicated by white crosses in c and e; fitting parameters (inserts) and residuals (bottom panels) are also shown; i) amplitude of the longest lifetime component histograms before and after Cu‐GTSM incubation; j) average lifetime and k) brightfield image of cells with fluorescence image overlayed; l) statistics of average lifetime change before and after treatment recorded for 12 cells, over 3 independent biological repeats.

The resulting intensity‐weighted average lifetime (τ_avg_=2.06 ns, τ_1_=1.1 ns, τ_2_=2.6 ns, α_1_=0.41, α_2_=0.59, χ^2^=1.18; see Figure S9c and compare to cell‐free data in Table S1) in lysosomes indicates that the probe is partially bound to copper(I). To confirm further the ability of **FLCS1** to image copper(I) in cellulo, we artificially increased the cellular concentration of copper(I) using Cu‐GTSM. This is a well‐established reagent that upon cellular uptake undergoes a bio‐reduction process releasing copper(I) inside a cell.^[39]^ Comparing the FLIM images of cells before and after treatment, the most suitable fitting model remains a biexponential decay. The fitting (Figure S9a,b) shows significant changes in the amplitudes of the fluorescence lifetimes following the addition of Cu‐GTSM, without significant changes to the distribution of the short and long lifetime components (Figure S9). To reduce the uncertainty in the determination of the fitting parameters, the two lifetime components were fixed at τ_1_=0.7 ns and τ_2_=2.7 ns for copper(I) treatment analysis. This did not influence the goodness of fit parameter (χ^2^) distribution (see Figure S13) but allowed us to clearly visualise the changes of the amplitudes of two lifetimes before and after the treatment (see Figure S12).

Consistent with the release of additional copper(I), an upwards shift in the average fluorescence lifetime (or, in other words, an upshift in the longer component amplitude, Figure S12a,b) of the **FLCS1** probe was observed upon treating SH‐SY5Y cells with Cu‐GTSM (5 μM for 20 minutes) (see Figures 3 b and 3e). This increase in the amplitude of the longer lifetime component α_2_ (see in Figure S12a,b), rather than its lifetime, is consistent with an increase in the fraction of the bound copper(I) seen by FLIM in cells following the treatment. It should be noted that the fluorescence intensity did not undergo significant changes following Cu‐GTSM treatment (see Figures 3 d and 3f), confirming that FLIM is a superior technique compared to intensity‐based imaging, enabling us to monitor small changes in copper(I) abundance in cells. As controls, treatment of cells with DMSO as well as the repeated exposure of the cells cultured in the presence of **FLCS1** to the laser irradiation did not lead to significant changes in fluorescence lifetime compared to cells treated with Cu‐GTSM (see Figure S14 and S15).

In conclusion, the new BODIPY‐based **FLCS1** probe shows excellent in vitro selectivity for copper(I) ions over all other physiologically abundant metal ions. Coordination of copper(I) to **FLCS1** not only leads to a large switch‐on of the probe's fluorescence intensity, but also to a significant increase in its fluorescence lifetime. Incubation of **FLCS1** with live SH‐SY5Y cells showed that at concentrations used for imaging the probe is non‐toxic and largely localised in lysosomes. Our studies are consistent with the direct detection of copper(I) in lysosomes of live cells confirming previous reports indicating that lysosomes have high concentration of this metal ion. Our data is also consistent with direct detection of an increase of copper(I) concentration in live cells via FLIM upon treatment with copper(I) releasing agent Cu‐GTSM.

## Conflict of interest

The authors declare no conflict of interest.

## Supporting information

As a service to our authors and readers, this journal provides supporting information supplied by the authors. Such materials are peer reviewed and may be re‐organized for online delivery, but are not copy‐edited or typeset. Technical support issues arising from supporting information (other than missing files) should be addressed to the authors.

Supporting Information
