# Supplementary material for: Selective Detection of Cu+ Ions in Live Cells via Fluorescence Lifetime Imaging Microscopy
Source: Angew Chem Int Ed Engl. 2021 Sep 17;60(43):23148–53. doi: 10.1002/anie.202109349 (PMC8596571; doi:10.1002/anie.202109349)
Supplement: Supplementary file 1 — Supporting Information [file ANIE-60-23148-s001.pdf]

Angewandte Chemie
Eine Zeitschrift der Gesellschaft Deutscher Chemiker

Supporting Information

Selective Detection of Cu^{+} Ions in Live Cells via Fluorescence Lifetime Imaging Microscopy

Martin Priessner, Peter A. Summers, Benjamin W. Lewis, Magdalena Sastre, Liming Ying, Marina K. Kuimova, and Ramon Vilar**

anie_202109349_sm_miscellaneous_information.pdf

Supporting Information

Table of Contents

Synthetic Materials and Methods. 2

Synthesis and characterization 2

Figure S1. MALDI-TOF spectrum of FLCS1 4

Figure S2. ^1H NMR spectrum (in $CDCl_3$) FLCS1 4

General protocols and instrumentation 5

Supporting figures and tables: 8

Figure S4. Job plot. 8

Figure S5. Reversibility fluorescence emission experiment. 8

Figure S6. Bi-exponential lifetime fitting comparison. 9

Table S1. TCSPC titration parameters. 10

Figure S8. Visual viability inspection. 11

Figure S9. FLIM imaging with variable lifetime values. 12

Figure S10. Time resolved traces recorded for FLCS1 (1 µM) in the presence of Cu^+ (3 µM) in the presence of various H_2O concentration, changing viscosity and pH 13

Figure S11. Lysosome acidification inhibition with bafilomycin A1. 13

Figure S12. FLIM imaging with fixed lifetime values. 14

Figure S13. FLIM χ^2 comparison. 14

Figure S14. FLIM image analysis 15

Figure S15. FLIM change analysis. 15

References 16

Author Contributions 16

Synthetic Materials and Methods.

All chemical reagents and solvents for synthesis were purchased from commercial suppliers and were used without further purification. All moisture or oxygen sensitive reactions were carried out under a nitrogen atmosphere. The composition of solvent mixtures is given by volume ratio (v/v). Analytical and preparative thin layer chromatography was performed using Merck 60 F254 silica gel with 0.25 mm and 2 mm thickness, respectively. Flash chromatography (FC) was performed using Merck Kieselgel 60 at rt under a positive pressure. ^{1}H NMR spectra were collected in $CDCl_3$ or DMSO-d_6 (Cambridge Isotope Laboratories, Cambridge, MA) at 25 °C on a Bruker AV-300 or AV400 spectrometers. Chemical shifts (δ) are given in ppm and coupling constants in Hz. Notation for the ^{1}H-NMR spectral splitting patterns include singlet (s), doublet (d), triplet (t), quartet (q), quintet (qui), broad (br) and multiplet/overlapping peaks (m). Matrix-assisted laser desorption/ionization MALDI analyses were carried out with Thermo Scientific CHCA MALDI Matrix. All compounds shown in the reaction schemes below (except for **5** and **FLCS1** which are new) were synthesized following previously reported procedures.[1],[2]

Synthesis and characterization

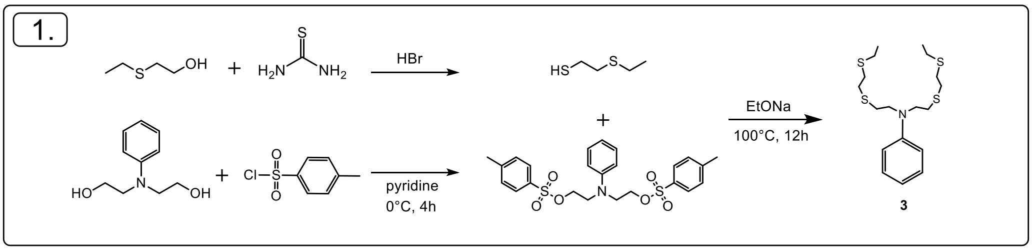

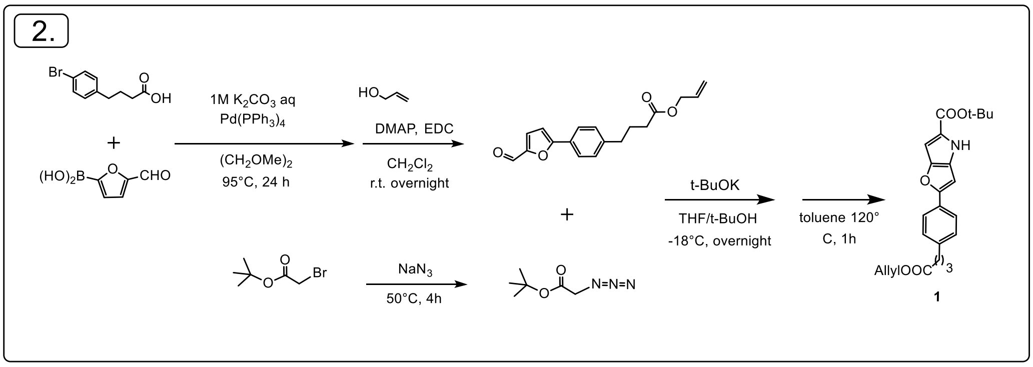

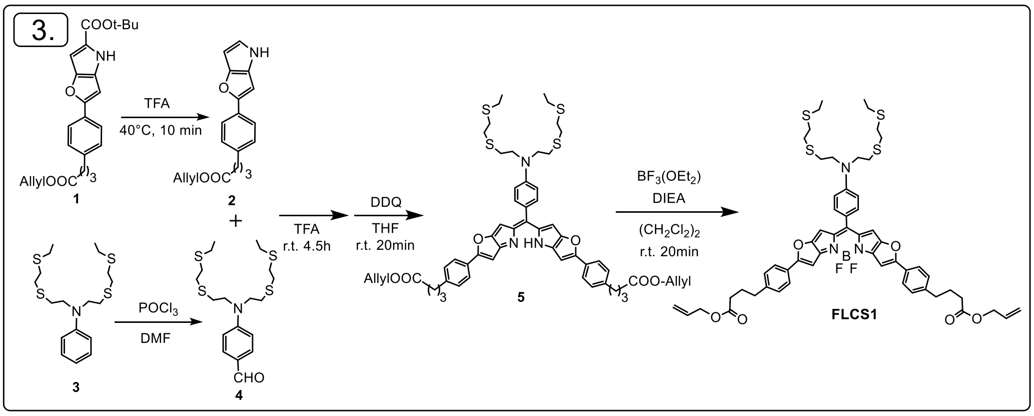

Synthesis of divinyl 4,4'-((11-(4-(bis(2-((2-(ethylthio)ethyl)thio)ethyl)amino)phenyl)-5,5-difluoro-5H-4l4,5l4-furo[2',3':4,5]pyrrolo[1,2-c]furo[2',3':4,5]pyrrolo[2,1-f][1,3,2]diazaborinine-2,8-diyl)bis(4,1-phenylene))dibutyrate (FLCS1)

Compound **1** (500 mg, 1.22 mmol, 2 eq.) was dissolved in acetone and transferred into a Schlenk flask. The solvent was carefully evaporated under reduced pressure in a Schlenk line. After that, **1** was redissolved in trifluoroacetic acid (5 ml) and the mixture was heated up under nitrogen atmosphere to 40 °C. After 10 minutes when the starting material was consumed, the mixture containing compound **2** was cooled down to room temperature and compound **4** (0.254 g, 0.61 mmol, 1 eq.) was added to the reaction mixture. After 15 minutes the solvent was evaporated under reduced pressure in a Schlenk line and the residue was redissolved in THF (8 ml). 2,3-Dichloro-5,6-dicyano-p-benzoquinone, DDQ (276 mg, mmol, 2 eq.) was separately dissolved in THF (1 ml) and slowly added with a syringe through a septum into the Schlenk flask. The reaction was stopped after 20 minutes by adding water; the crude product was extracted with CH_2Cl_2. The organic layer was washed with brine and water, dried over Na_2SO_4 and evaporated. The mixture was partially purified via flash column chromatography (eluent: n-Hexane/$CHCl_3$ = 40/60; solid phase: alumina) to remove the remaining DDQ from the reaction mixture.

The remaining solid containing crude compound **5** was dissolved in 1,2-dichloroethane under nitrogen; N, N-diisopropylethylamine, DIEA (0.83 ml, 0.61 g, 4.7 mmol, 7.8 eq.) was added and the reaction mixture heated-up to 60°C. Then $BF_3{\cdot}Et_2O$ (0.83 ml, 0.95 g, 6.7 mmol, 11 eq.) was added which led to an instant colour change from green to very dark blue. After 25 min toluene was added to the reaction mixture and a liquid-liquid extraction with water was carried out. The organic phase was again washed with brine and water and the solvent was evaporated. The final purification steps consisted of passing the product through a silica flash chromatography column (eluent: n-Hexane/EA = 100/00 to 80/20) followed by a preparative thin layer chromatography (eluent: n-Hexane/EA = 66/33); for both purification steps the silica used was firs treated with 0.1% triethylamine in hexane. The product obtained from column chromatography, was dissolved in acetone and deposited on the TLC plate with a pipette. The product band was scratched off from the TLC and extracted with MeOH; the solvent was removed under reduced pressure to yield pure **FLCS1**, which is a dark blue compound (21.7 mg, 3.3 % yield). A sample of the compound was used to prepare a 10 mM stock solution in DMSO and stored at -18°C. For all further spectroscopic measurements, this sample was diluted to 1 mM and 20 μM which served as a stock solution for the photophysical characterisation and for the cell experiments respectively.

^{1}H-NMR ($CDCl_3$, 400 MHz): δ 1.31 (t, 6H, J = 7.4 Hz), 2.03 (qui, 4H, J = 7.5 Hz), 2.41 (t, 4H, J = 7.4 Hz), 2.63 (q, 4H, 5.7 Hz), 2.74 (t, 4H, J = 7.6 Hz), 2.91 - 2.78 (m, 12H), 3.70 (t, 4H, J = 7.5 Hz), 4.61 (d, 4H, J = 5.7 Hz), 5.27 (d, 2H, J = 10.4 Hz), 5.35 (d, 2H, J = 16.9 Hz), 5.37 (d, 2H, J = 16.9 Hz), 6.00 - 5.88 (ddd, 2H, J = 5.7 Hz, 10.5 Hz, 17.1 Hz), 6.46 (s, 2H), 6.79 (d, 2H, J = 8.9 Hz), 7.00 (s, 2H), 7.30 (d, 4H,J = 8.2 Hz), 7.53 (d, 2H, J = 8.8 Hz), 7.77 (d, 4H, J = 8.3 Hz).

^{13}C-NMR ($CDCl_3$, 400 MHz): δ 14.9, 26.2, 26.3. 29.3, 29.5, 31.9, 32.6, 33.5, 35.1, 51.6, 65.1, 95.2, 103.5, 111.2, 118.3, 122.7, 125.5, 127.9, 129.2, 132.2, 132.8, 139.2, 143.7, 148.2, 148.6, 153.5, 167.1, 172.3.

MALDI: m/z calcd. for $C_{57}H_{64}BF_2N_3O_6S_4$: 1064.20 found: 1064.40 [M].

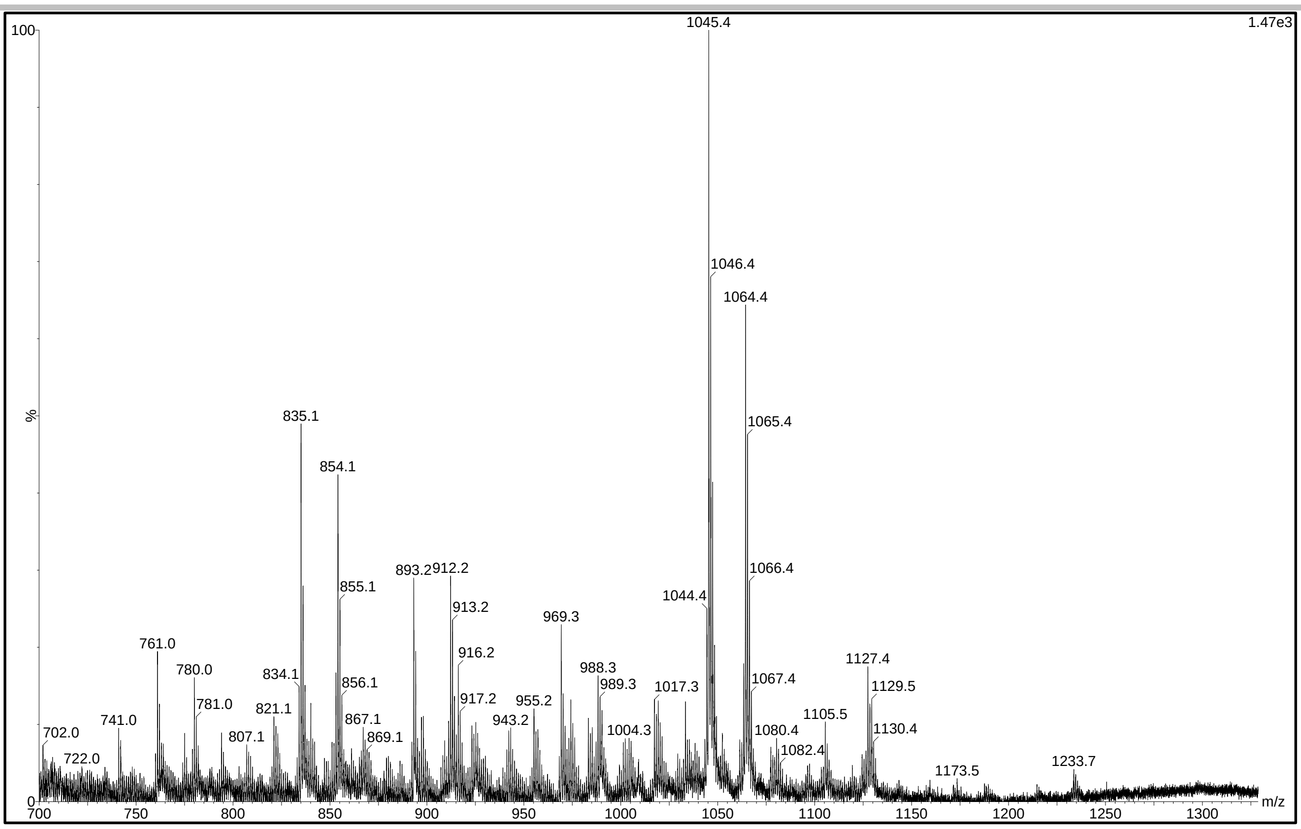

Figure S1. MALDI-TOF spectrum of **FLCS1**

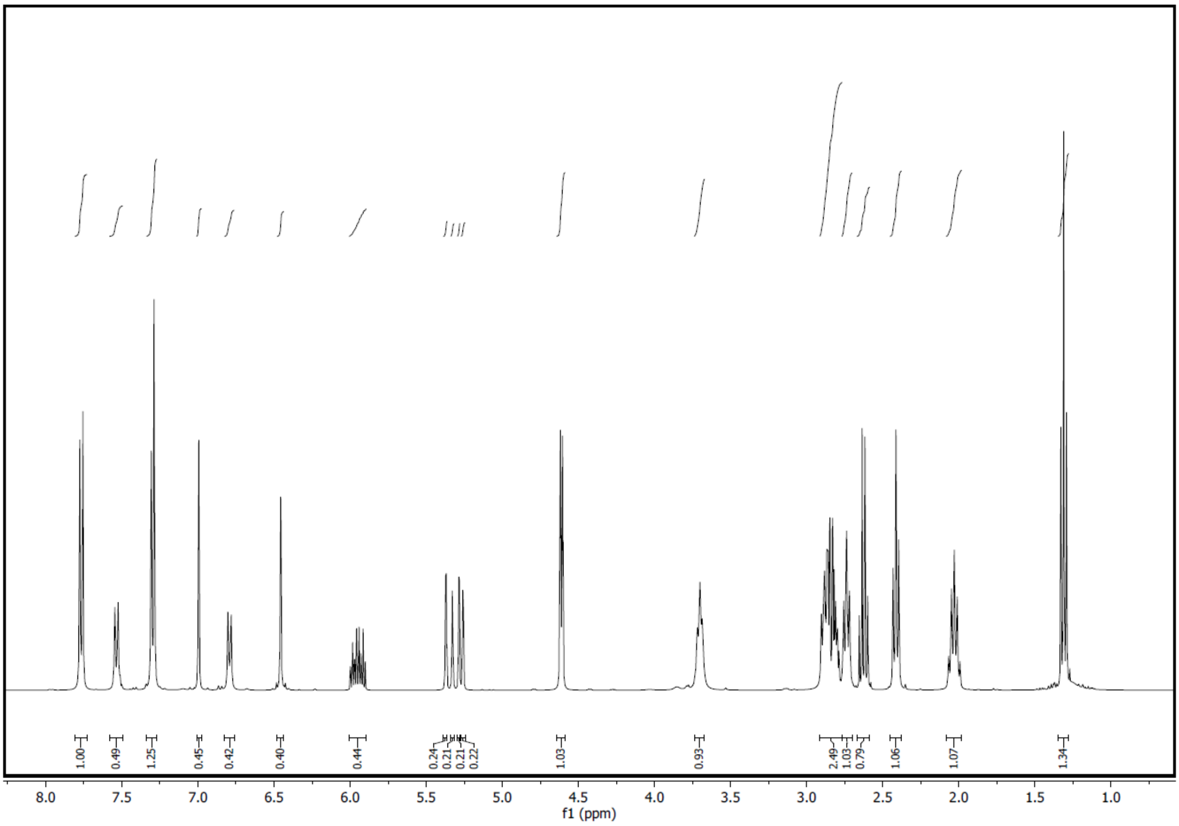

Figure S2. ^{1}H NMR spectrum (in $CDCl_3$) **FLCS1**

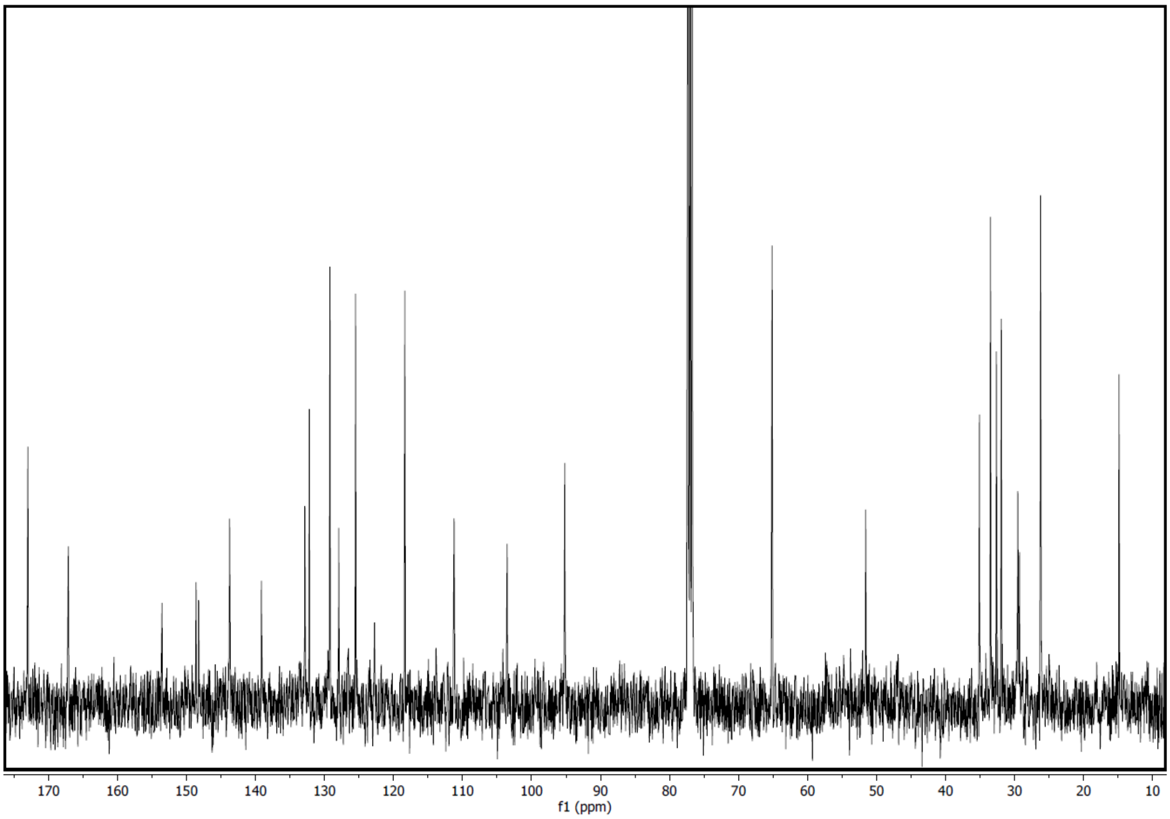

Figure S3. ^{13}C NMR spectrum (in $CDCl_3$) **FLCS1**

General protocols and instrumentation

UV/vis spectra were recorded in a Perkin-Elmer spectrometer. Emission spectra were obtained on Varian Cary-Eclipse or Perkin-Elmer fluorescence spectrometers. Time-resolved fluorescence decay traces were obtained using a TCSPC DeltaFlex Lifetime System using the 635-nm NanoLED-02B R excitation source (HORIBA Scientific Ltd, FWHM <200 ps). The detection system consisted of a PPD Series Picosecond Photon Detection Modules (HORIBA Scientific Ltd). A long-pass filter (645 nm long pass) was used in the detection channel to avoid light scattering from the excitation source in fluorescence decays. Signal intensity was at least 10,000 counts in the peak maximum. A neutral density filter was used for the instrument response function (IRF) measurements using a Ludox solution, detecting emission at the excitation wavelength. The TCSPC, absorption and emission measurements were performed in 1 cm × 1 cm quartz cuvettes with septum (3.5-mL volume, Starna, Atascadero, CA) or 0.35 cm × 1 cm quartz cuvettes (Cole-Parmer). The titration experiments were all performed under N_2 atmosphere by supplying positive nitrogen pressure to the cuvette with a nitrogen balloon. To account for possible delays in the fluorescence increase of the probe upon binding to Cu(I), the sample was stirred with a small stirrer bar in the dark before measuring each titration point by TCSPC and in fluorescence titrations. Fluorescence quantum yields were determined by using to Alexa 647 in water as a standard ($\Phi_f = 0.33$).

Preparation of metal ion solutions for spectroscopic experiments

For the spectroscopic fluorescence and absorbance experiments MeOH was degassed by purging with N_2 for 2 hours before the experiment to avoid oxidation of the copper(I) salt. During the experiment it was kept under N_2 atmosphere using a nitrogen balloon. 10 mM **FLCS1** stock solution was further diluted to 1 mM in DMSO. 37.3 mg of the Cu(I) metal Tetrakis(acetonitrile)copper(I) hexafluorophosphate $[Cu(MeCN)_4][PF_6]$ was dissolved in 5 mL degassed MeOH resulting in a 20 mM Cu(I) stock solution. This stock was then further diluted to 1 mM and 0.1 mM for the titration experiment. For the selectivity experiments with the different competing metals, 100 mM stock solutions of $CaCl_2$, NaCl, $MgCl_2$, $ZnCl_2$ and 1 mM stock solutions of KCl, $CoCl_2$, $FeSO_4$, MnBr, NiAc, and $CuSO_4$ in H_2O were prepared and 20 µl of each was added to a 1 ml methanolic solution of FLCS1 (1 µM).

Determination of Dissociation Constant

To determine the dissociation constant (K_d) a methanolic solution of **FLCS1** (1 μM) containing thiourea (400 μM) as competitive ligand. To calculate the available copper the stability constants for thiourea binding were used taken from the literature: $\beta_{12} = 2.0 \times 10^{12}$, $\beta_{13} = 2.0 \times 10^{14}$, $\beta_{14} = 3.4 \times 10^{15}$.[3] For this titration the copper(I) stock solution was further diluted to 1 μM (see values in caption of Fig. S4b). The fluorescence spectra was collected by exciting at 610 nm and the emission was integrated from 620 to 750 nm. The binding affinity was calculated following literature procedures [4,5] using the Benesi–Hildebrand plot (see Fig. S4c) with the equation: $(F - F_{min}) = \Delta F = [Cu^+](F_{max} - F_{min})/(K_d + [Cu^+])$ where F is the observed fluorescence, F_{max} is the fluorescence for the **FLCS1**-Cu^+ complex, and F_{min} is the fluorescence for the copper-free probe. When plotting the 1/△F against 1/[Cu^+] the linear relation equation (Y= A + BX) was used to calculate K_d from B/A.

Time-correlated single photon counting data fitting

Traces from the TCSPC were fitted by iterative reconvolution to the equation $I(t) = I_0(\alpha_1 e^{-\frac{t}{\tau_1}} + \alpha_2 e^{-\frac{t}{\tau_2}})$ where α_1 and α_2 are variables (in %), with $\alpha_1+\alpha_2$ normalized to unity. The fractional contribution to the steady-state emission is calculated from the equation $f_i = \alpha_i\tau_i / \sum_j \alpha_j\tau_j$. The τ_1 and τ_2 in the *in vitro* titration were fixed to 0.39 ns and 3.08 ns based on the global fit over the whole titration data. To account for the differences in the emission wavelength between the IRF and decay, a prompt shift was included in the fitting. The goodness of fit was judged via a weighted residuals plot by consideration of the deviations from the model.

Cu-GTSM preparation

The Cu-GTSM was prepared fresh before every use by mixing stock solution of 10 mM GTSM dissolved in DMSO with the same volume of 10 mM $CuCl_2$ dissolved in H_2O. The compound formed instantly after thorough mixing by pipetting the two solutions which could be observed by a colour-change from transparent to dark orange. The compound was then used within a few hours. The 250 μl of DMEM containing the treatment agent (10 μM Cu-GTSM) resulting in a chamber concentration of 5 μM, was added to chambers for 20 minutes following FLIM measurements of the “after treatment” state.

Preparation and staining of cell cultures

SH-SY5Y cells were cultured in Dulbecco’s Modified Eagle Medium (DMEM, Invitrogen, Carlsbad, CA) supplemented with 10% Fetal Bovine Serum (FBS, Invitrogen), glutamine (2 mM), and penicillin/streptomycin (50 μg/mL, Invitrogen). All cells were grown in a 5% CO_2 incubator at 37 °C. The cells were plated and grown on 8-well chamber slides (LabTekII Chamber Coverglass) in 250 μL of culture media at a plating density of 25,000 cells per well and allowed to grow for 24 h.

Next the media was changed to media containing lipofectamine 2000 (2μl/ml) and **FLCS1** (60 nM). The cells were incubated with the dye for 24 h. Prior to imaging the cell media was changed back to DMEM with 10% FBS (250 μl/well). The lysosome stain Lysotracker Green (λ_{ex} = 500 nm; λ_{emis} = 510–530 nm; Invitrogen, ThermoFisher) was incubated with cells at 50 nM for 5 min prior to 3 washing cycles in DMEM and subsequent image acquisition on a confocal microscope (Leica SP5) with 40x or/and 63x magnification 1.4 NA oil objective.

For FLIM imaging the coverslide was mounted in a microscope chamber heated by a circulating thermostat (Lauda GmbH, E200) with feedback control of temperature and 0.2 °C precision. Cells were measured by FLIM, as described below, in the ‘before’ and ‘after’ treatment states.

Bafilomycin A1 treatment experiment

SH-SY5Y cells were grown in DMEM with 10% FBS with and added penicillin and streptomycin at 37°C. The healthy cells were plated on LabTek II coverslides at a seeding density of 20.000 cells per well and let grow for 48h more hours in an incubator. Then the media was changed to media containing 0.2% lipofectamine and 60 nM FLCS1 and the cells were incubated for another 24h. After that the media was changed back to grow media and the after a calming period of 1 hour the cells were imaged in their “before treatment” state on a Leica SP7 confocal microscope at 630 nm excitation and a laser power of 0.1 %. The location of the cells was precisely recorded by measuring the coordinate distances of a set reference point of a cover glass impurity at the well corner. Then

the cells were treated with bafilomycins A1 (30 nM) in DMEM and was incubated for 24 hours. For the second time point the same cells were searched based on the recorded coordinates and measured with the same microscopy settings as before.

For the control comparisons the control cells were treated with DMSO (0.01%) and also incubated for 24 hours. After the incubation time images were taken with the same settings as for the bafilomycin A1 treated cells.

FLIM imaging experiments

FLIM imaging with cells incubated with **FLCS1** was performed on an inverted confocal laser scanning microscope (Leica, SP5 II) with a ×63 (NA 1.4) water immersion objective and a pulsed diode laser (Becker & Hickl GmbH, 630 nm, 20 MHz) as an excitation source. The emission (640–750 nm) was collected using a cooled Becker&Hickl HPM100-40 hybrid detector, and TCSPC was performed by a SPC-150 Becker&Hickl module. FLIM images of 512 × 512 pixels were obtained, and the images were analysed in the SPCImage software (Becker&Hickl, Germany) using a bi-exponential decay model with τ_1 and τ_2 fixed at 0.7 ns and 2.7 ns respectively. The regions of interests (ROIs) for analysis were manually selected, to coincide with bright fluorescence of the dye. The scatter parameter was kept variable and the shift was fixed to the overall average shift of all ROIs. Appropriate bin sizes were chosen (3 × 3 or 4 × 4 circular bin) to ensure a peak count of at least 100 for accurate fitting of bi-exponential traces and a sum count threshold of 4000 was used to exclude dye traces in the cytosol or other not relevant regions. A pseudo-colour scale was assigned to each fluorescence lifetime, amplitude, and the goodness of fit χ^2 values (red for small values and blue for large values) to visualise the corresponding maps. The IRF was obtained by measuring SHG signal from the reflection of urea crystals on a glass cover slide. Cellular images were acquired using the same settings, and the acquisition times was ca. 15 s - 30 s (live cells) depending on the brightness of the dye. The decays in the region corresponding to the lysosomes of different cells were selected and analysed by hand (ca. 15 different cells in 7—8 images, to accumulate good statistics). The fitting of the FLIM images was performed in SPCImage software (Becker-Hickl) using the weighted least squares method and reconvolution algorithm for finding the best fit. Goodness of fit was judged by the χ^2 value and randomness of the residuals plot. Based on the TCSPC experiments the decay models of the fluorescence dye were judged to be a bi-exponential and were calculated using the following equation: $\mathrm{I(t)} = \sum_{i=1}^{n} \alpha_i e^{-t/\tau_i}$ where *I* is fluorescence intensity, *t* is time, and α_i is the amplitude (in fractions of 1), and τ_i is the fluorescence lifetime of the bi-exponentially decaying components. The calculation of the mean fluorescence lifetime was according to the equation: $\tau_{avg} = \frac{\sum \alpha_i \tau_i^2}{\sum \alpha_i \tau_i}$.

pH/viscosity/H_2O-effect experiments

The TCSPC pH measurements were carried out in a methanolic solution of FLCS1 (1 µM). The pH was calibrated using diluted HCl and NaOH solutions. For the viscosity experiment the probe was dissolved in different ratios of MeOH and glycerine and for the water-effect on the lifetime the solvent ratios were altered between water and MeOH. All the measurements were carried out with excess copper(I) (3 µM).

Supporting figures and tables:

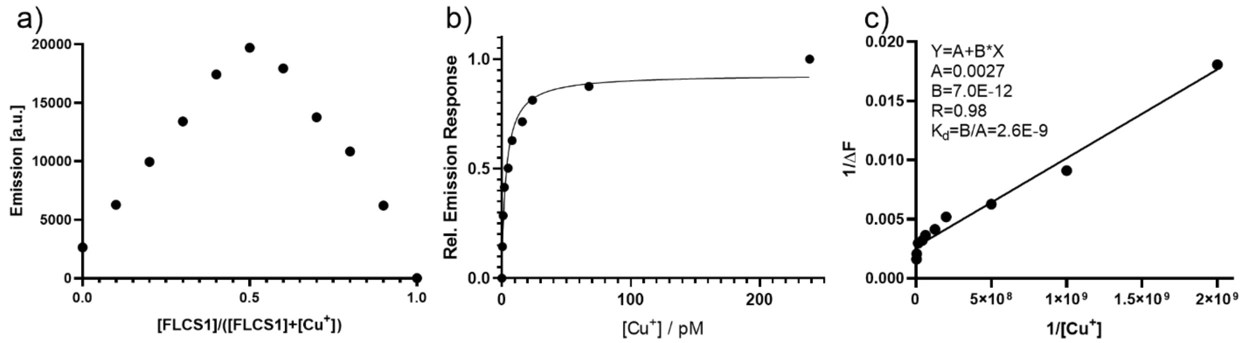

Figure S4. a) Job plot. **FLCS1** with Cu(I) at concentrations of 2.5-0 μM for **FLCS1** and 0-2.5 μM for Cu(I). The total combined concentration of **FLCS1** and Cu(I) were kept constant at 2.5 μM. Excitation wavelength was 610 nm and collection-range 615-700 nm. The spectra were acquired in MeOH. The maximum fluorescence response at 1.25 μM fraction of FLCS1 indicates the formation of a 1:1 Cu(I):**FLCS1** complex. **b) Normalized binding affinity titration for Kd determination.** Response of **FLCS1** (1 μM) in MeOH containing thiourea (400 μM) and free Cu^+ ions (0, 0.49, 0.99, 1.98, 4.97, 7.95, 15.91, 23.86, 67.61, 238.63 pM). c**) Benesi–Hildebrand plot** of competition titration (from b)) resulting in a calculated K_d value of $2.6*10^{-9}$ (not directly comparable to previously reported values due to the titration being done in different solvents).

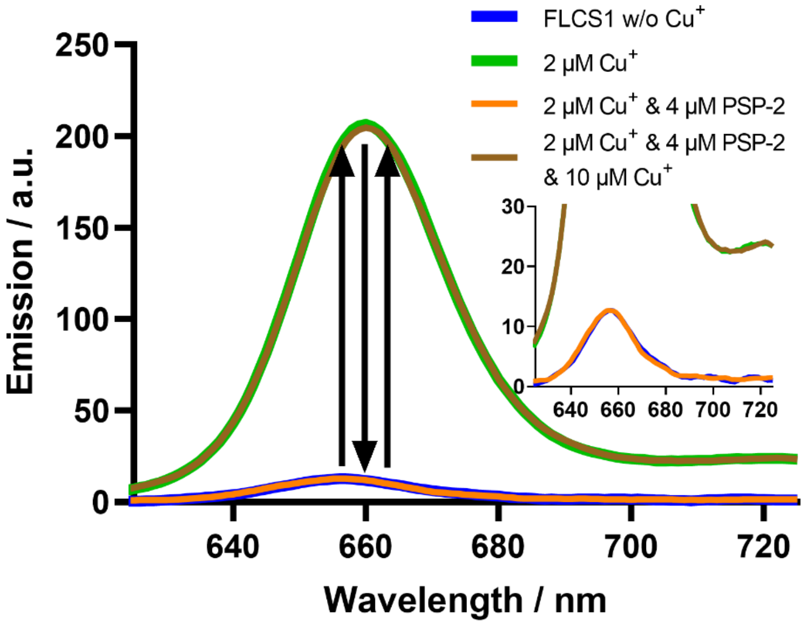

Figure S5. Reversibility fluorescence emission experiment. FLCS1 (1 μM) in MeOH was repeatedly measured before and after Cu^+ (2 μM) addition, followed by addition of PSP-2 (4 μM) and repeated addition of excess Cu^+ (10 μM) showing reversibility of the probe's response to copper(I) addition/depletion.

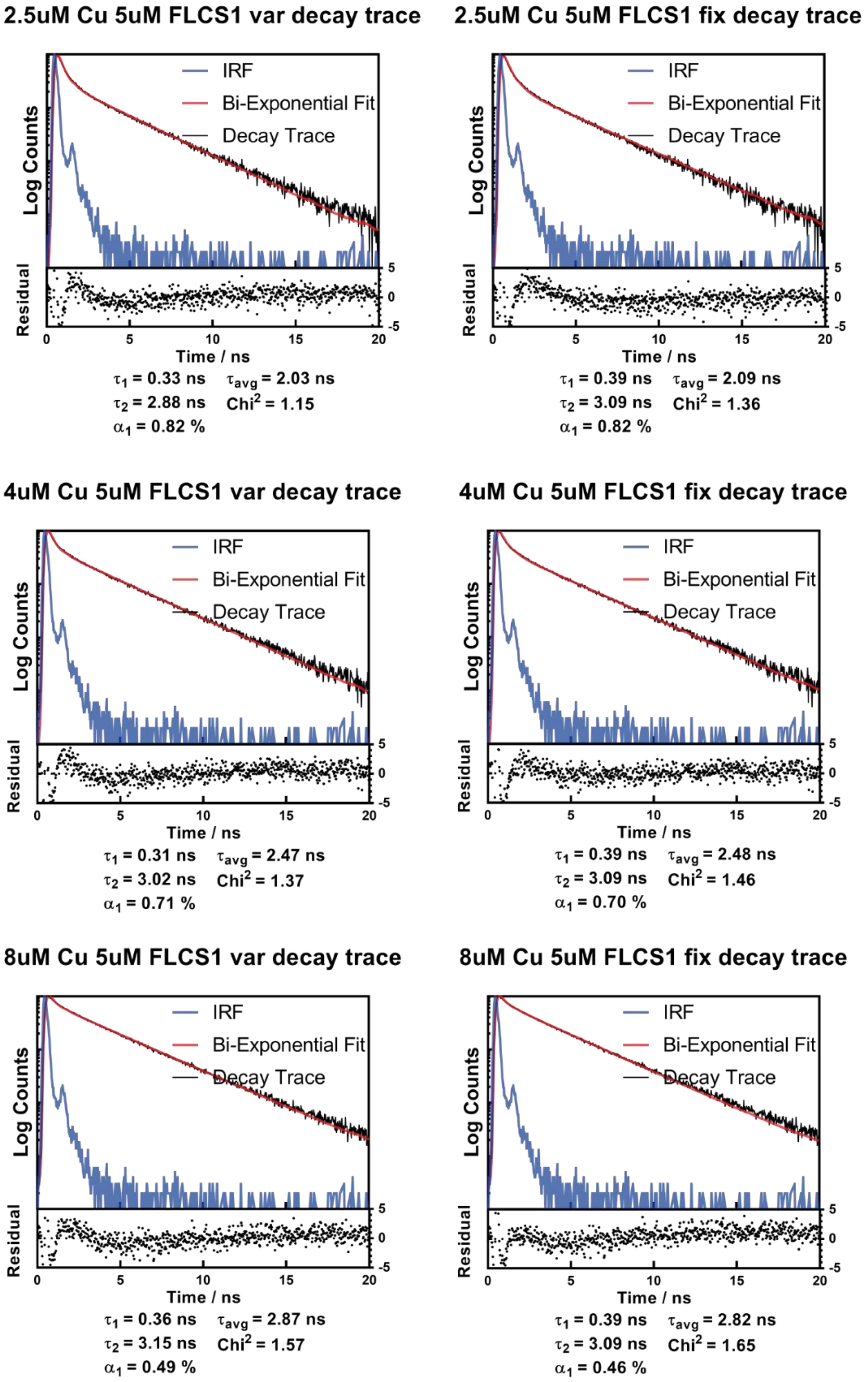

Figure S6. Bi-exponential lifetime fitting comparison. FLCS1 (5 μM) in with Cu^+ (2.5, 4, 8 μM) lifetime was measured and traces were fitted two exponentials with τ_1 and τ_2 variable (left) and fixed at τ_1 = 0.39 and τ_2 = 3.09 ns (right).

Table S1. TCSPC titration parameters. TCSPC fitting parameters of titration of Cu(I) to **FLCS1** (1 μM) in methanol; bi-exponential fit with fixed values for τ_1 = 0.39 ns and τ_2 = 3.09 ns.

	α1	α2	τ_{avg}	χ^2
**FLCS1 0.0 eq Cu(I)**	0.982	0.018	0.73	1.95
**FLCS1 0.1 eq Cu(I)**	0.957	0.043	1.09	1.6
**FLCS1 0.2 eq Cu(I)**	0.908	0.092	1.59	1.54
**FLCS1 0.3 eq Cu(I)**	0.897	0.103	1.67	1.37
**FLCS1 0.4 eq Cu(I)**	0.862	0.138	1.9	1.38
**FLCS1 0.5 eq Cu(I)**	0.823	0.177	2.09	1.33
**FLCS1 0.6 eq Cu(I)**	0.782	0.218	2.25	1.36
**FLCS1 0.7 eq Cu(I)**	0.741	0.259	2.37	1.44
**FLCS1 0.8 eq Cu(I)**	0.696	0.304	2.48	1.46
**FLCS1 0.9 eq Cu(I)**	0.652	0.348	2.57	1.49
**FLCS1 1.0 eq Cu(I)**	0.607	0.393	2.64	1.53
**FLCS1 1.2 eq Cu(I)**	0.531	0.469	2.75	1.63
**FLCS1 1.4 eq Cu(I)**	0.49	0.51	2.79	1.59
**FLCS1 1.6 eq Cu(I)**	0.461	0.539	2.82	1.64
**FLCS1 1.8 eq Cu(I)**	0.454	0.546	2.83	1.64
**FLCS1 2.0 eq Cu(I)**	0.459	0.541	2.82	1.52

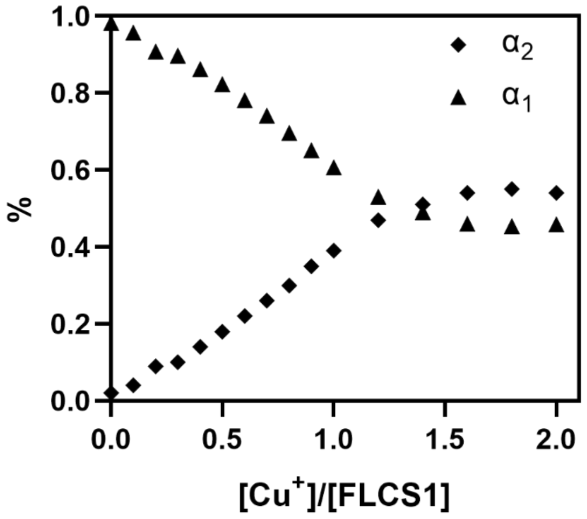

Figure S7. Titration amplitude trend. *In vitro* fluorescence lifetime titration results of a methanolic solution of **FLCS1** (1μM) with Cu^+ for the amplitude values at fixed values of τ_1 = 0.39 ns and τ_2 = 3.08 ns, obtained from the global fit of the whole dataset.

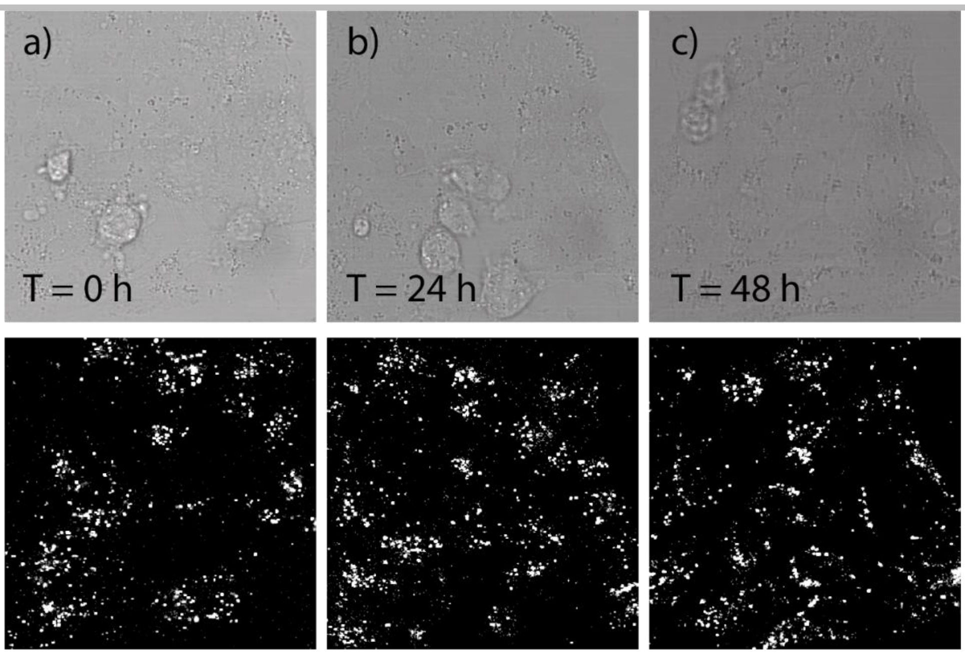

Figure S8. Visual viability inspection. Brightfield (top) and fluorescence image (bottom) of SH-SY5Y cells treated for 24 h with DMEM containing 60 nM **FLCS1** and 0.2% lipofectamine and 10% FBS and measured at timepoint a) 0 hours b) 24 hours and c) 48 hours after changing back to unsubstituted media. The cells show normal morphology (λ_{ex} = 635 nm, λ_{emis} = 645–770 nm).

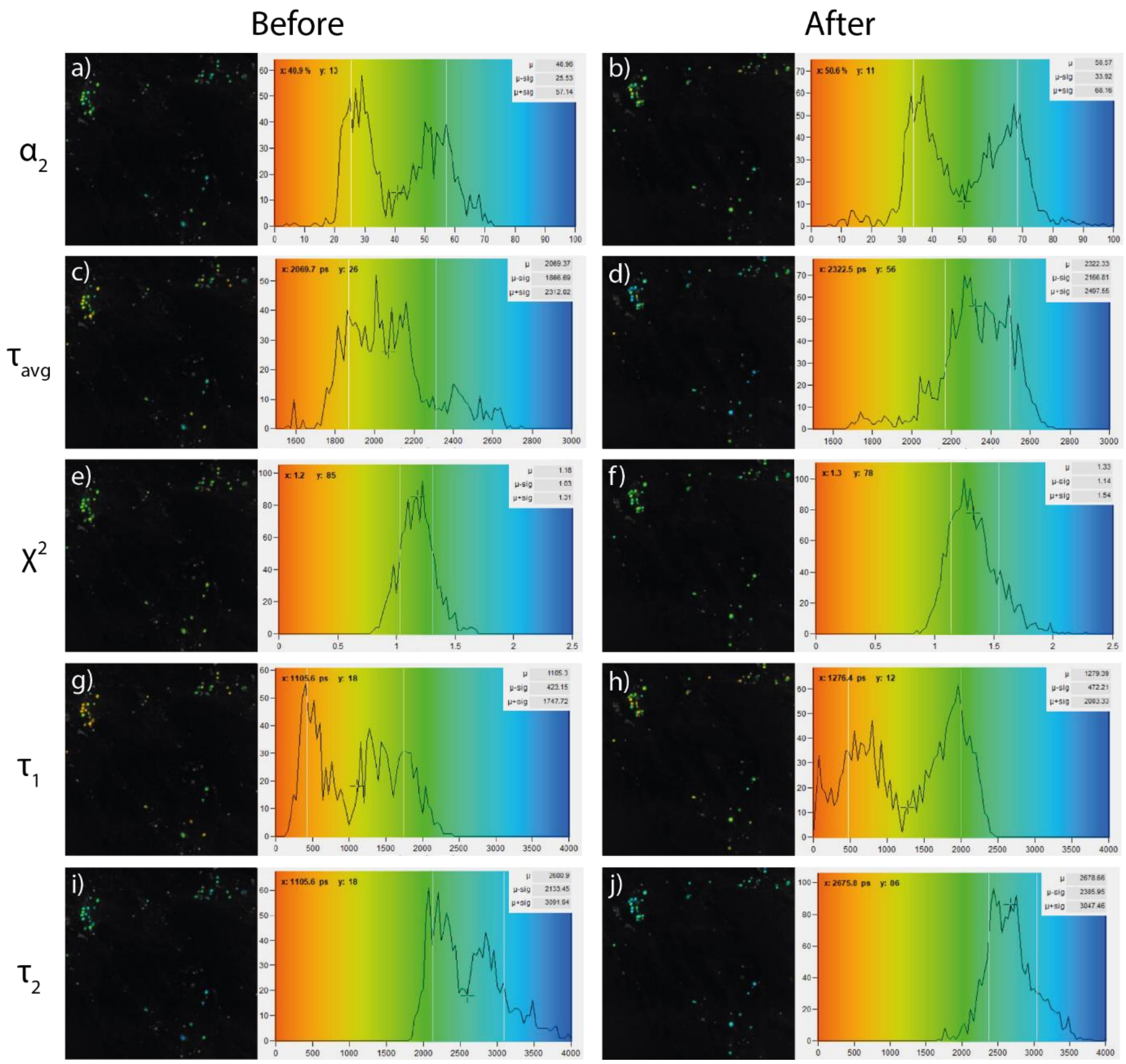

Figure S9. FLIM imaging with variable lifetime values. Example FLIM images and corresponding histograms of parameter distributions for **FLCS1** in SH-SY5Y cells showing a, b) α_2; c, d) τ_{avg}; e, f) χ^2; g, h) τ_1 and g, h) τ_2 obtained for the fitting protocol with freely variable values of τ_1 and τ_2, before (left) and after (right) 20 min treatment of cells with Cu-GTSM. Only small variations in τ_1 and τ_2 values were recorded before and after the treatment with Cu-GTSM, *i.e.* compare g) with h) and i) with j). Therefore, the reduce the uncertainty of the fitting parameters, the decision was taken to fix τ_1 and τ_2 values, see Figure S12 for the results. We note that the goodness of fit χ^2 is not affected by fixing the lifetime values. Also, our conclusions that the amplitude of the long component, α_2, is increased upon treatment with Cu-GTSM, is valid, irrespective of the fitting method, Figure S9 or Figure S12.

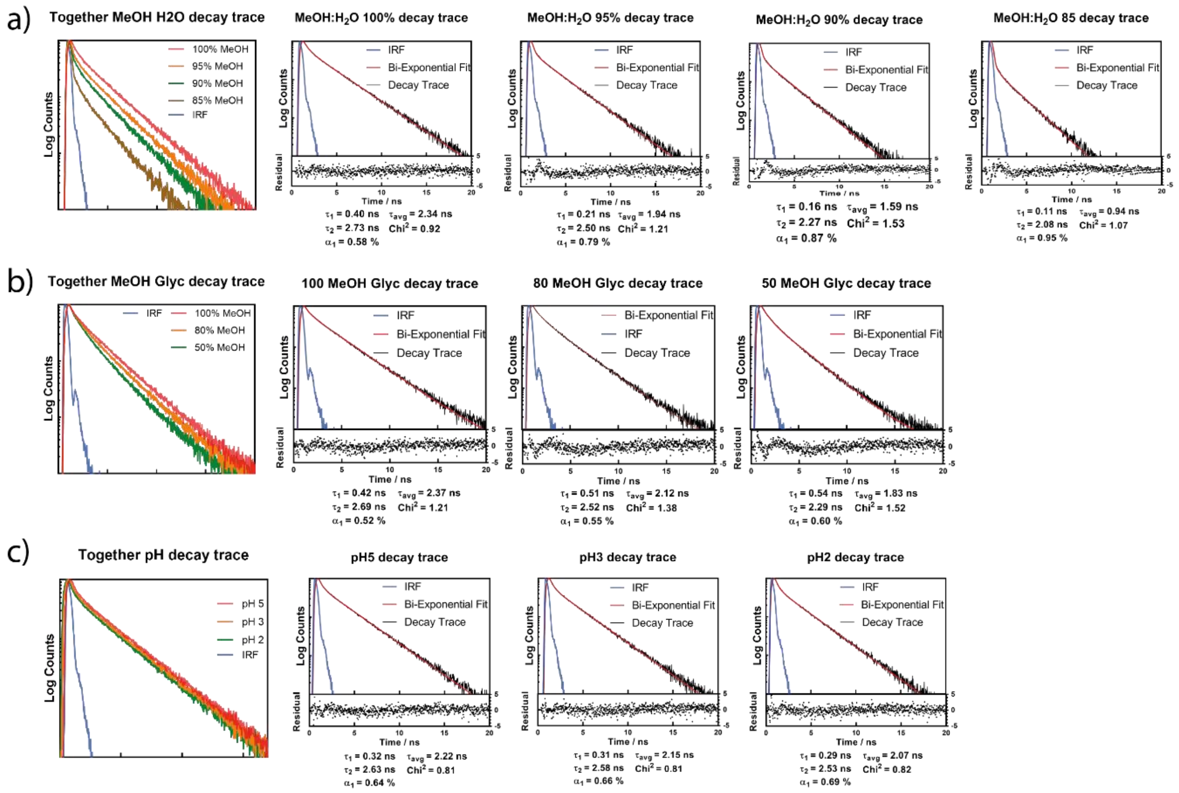

Figure S10. Time resolved fluorescence decay traces recorded for FLCS1 (1 μM) in the presence of Cu^+ (3 μM) in the presence of various environmental quenchers: H_2O, changing viscosity and pH; a) different MeOH:Glycerol ratios, increased [Glycerol] causes an increase in viscosity; b) different MeOH:H_2O ratios; c) different pH values. The pH was adjusted using dilute HCl and NaOH solutions. Average lifetimes were evaluated using bi-exponential fitting with variable τ_1 and τ_2 values. The decay traces, IRF, biexponential fit traces, residuals and fitting parameters are shown for each condition.

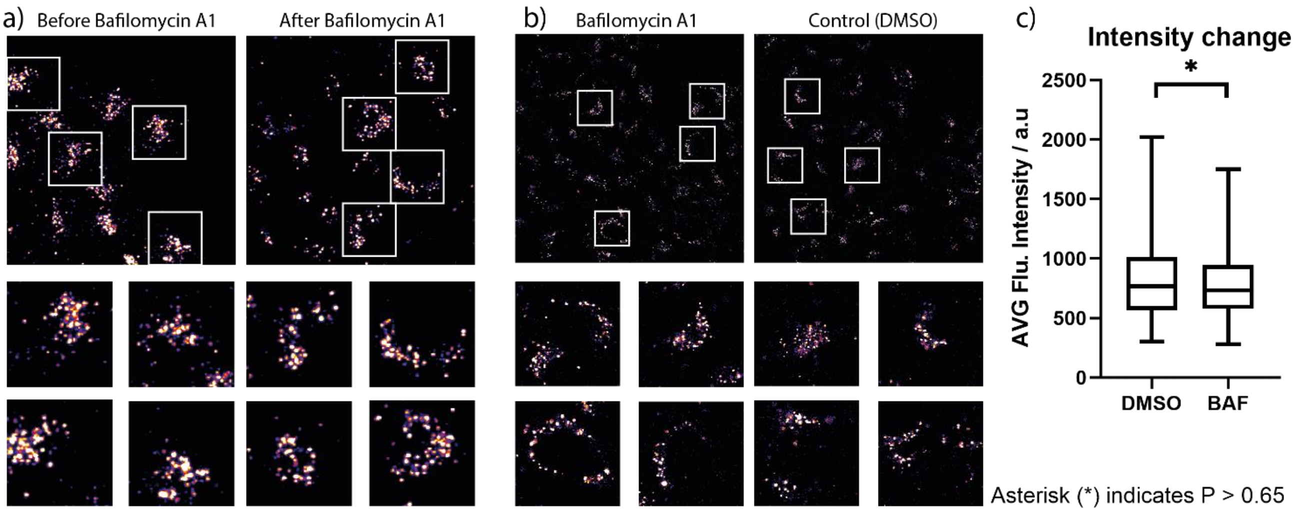

Figure S11. Lysosome acidification inhibition with bafilomycin A1. SH-SY5Y cells were treated for 24 h with DMEM containing 60 nM **FLCS1** and 0.2% lipofectamine and 10% FBS a) imaged before and after treatment with bafilomycin A1 (30 nM) for 24h; b) comparison of bafilomycin A1 (30 nM) treated cell compared to equivalent concentrations DMSO (0.01%) treated cells. c) Statistical student t-test of intensity comparing 48 and 41 cells treated with bafilomycin A1 and DMSO, respectively, showing no statistically significant change of lysosomal intensity signals.

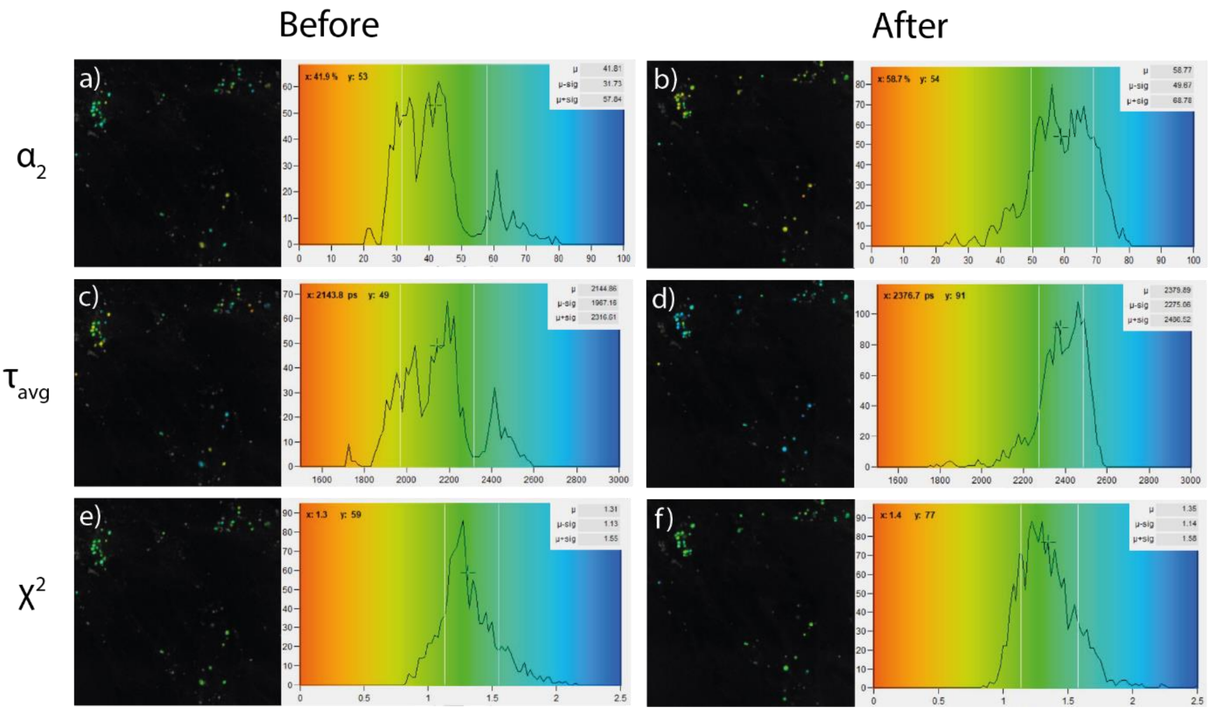

Figure S12. FLIM imaging with fixed lifetime values. Example FLIM images and corresponding histograms of parameter distributions for **FLCS1** in SH-SY5Y cells showing a, b) α_2; c, d) τ_{avg}; e, f) χ^2; obtained at fixed values for τ_1 =0.7 ns and τ_2 =2.7 ns before (left) and after (right) 20 min treatment of Cu-GTSM; the histogram distributions of each of the variable is also shown. Our conclusions that the amplitude of the long component, α_2, is increased upon treatment with Cu-GTSM, is valid, irrespective of the fitting method (with or without fixing of τ_1 and τ_2), *cf* Figure S9 and Figure S12.

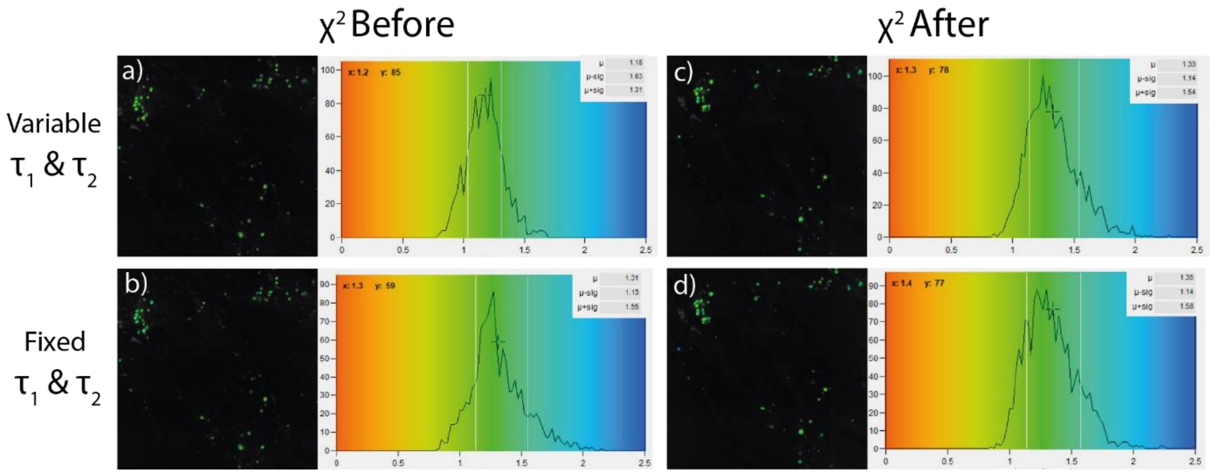

Figure S13. FLIM χ^2 comparison. Example FLIM images of **FLCS1** in SH-SY5Y cells showing χ^2 value with variable τ_1 and τ_2 values a) before and c) after; and for values fixed at τ_1 =0.7 ns and τ_2 =2.7 ns, b) before and d) after 20 min treatment of Cu-GTSM; histograms of χ^2 value distributions are also shown.

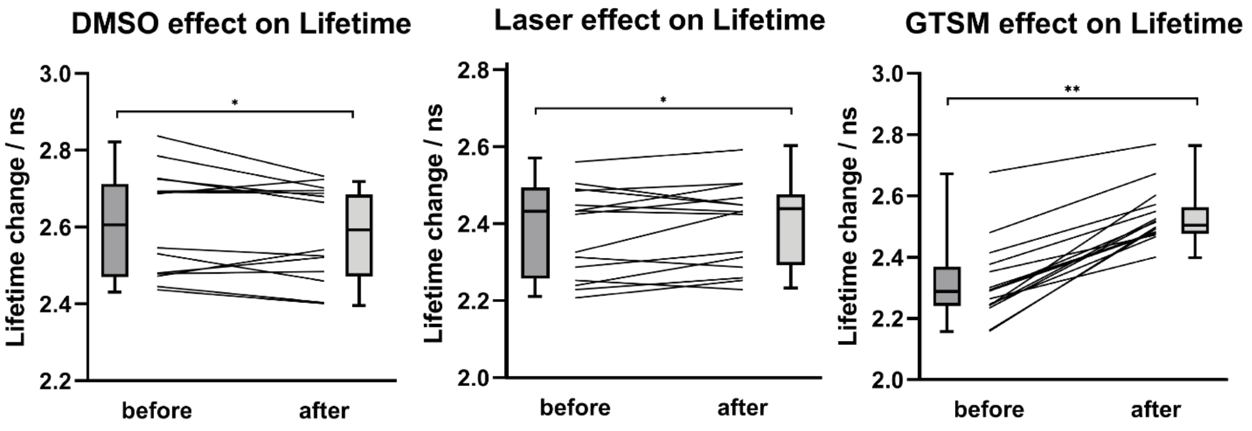

Figure S14. FLIM image analysis. Effect of 20 min incubation with 1% DMSO in DMEM (left), effect of repeated laser exposure (middle), effect of Cu-GTSM (2.5 μM) in DMEM on average fluorescence lifetime (τ_{av}) of **FLCS1** in SH-SY5Y cells (right). The average lifetime was calculated with fixed τ_1 =0.7 ns and τ_2 =2.7 ns values, as shown in Figure S12. * p > 0.5 shows no statistical significance ** p < 0.0005 shows statistical significance with student t-test.

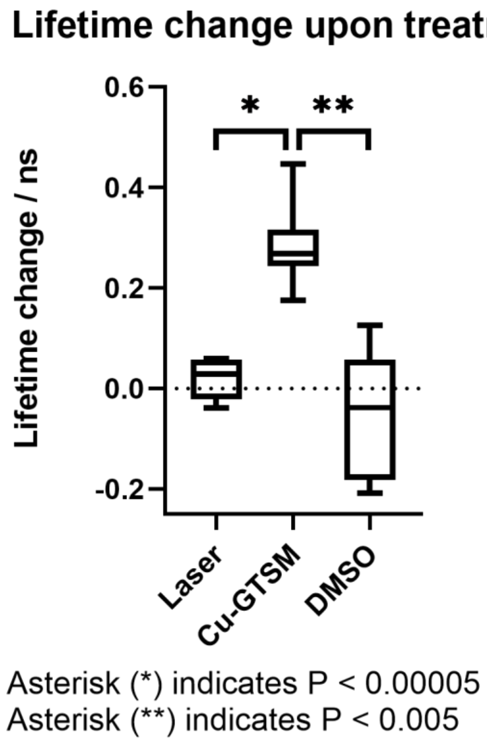

Figure S15. FLIM change analysis. The changes of average lifetime are displayed before and after laser exposure (left), Cu-GTSM treatment (middle) and DMSO treatment (right) showing statistical significance for observed changes with * p > 0.0005 and ** p < 0.00005 with student t-test.

References

[1] A. Matsui, K. Umezawa, Y. Shindo, T. Fujii, D. Citterio, K. Oka, K. Suzuki, *Chem. Commun.* **2011**, *47*, 10407–10409.

[2] J. Ishikawa, H. Sakamoto, H. Wada, *J. Chem. Soc.*, **1999**., 2, 1273-1279

[3] R. M. Smith, A. E. Martell, Critical Stability Constants (Plenum, New York) **1989**.

[4] X. Cao, W. Lin, W. Wan, *Chem. Commun.* **2012**, *48*, 6247–6249.

[5] S. C. Dodani, D. W. Domaille, C. I. Nam, E. W. Miller, L. A. Finney, S. Vogt, C. J. Chang, *Proc. Natl. Acad. Sci.* **2011**, *108*, 5980–5985.

Author Contributions.

M.P., M.K.K. and R.V. designed the study, analyzed the data and co-wrote the paper; M.P. and P.S. performed experiments and analyzed the associated data. B.L. provided advice and helped with the analysis of FLIM data; M.S. provided advice for the cellular studies. L.Y. provided advice on probe design and initial photophysical data.
